# Correction: Research on fire scenario analysis and emergency response strategies for L-shaped buildings using FDS

**DOI:** 10.1371/journal.pone.0352799

**Published:** 2026-06-29

**Authors:** Qin Zhang, Yuhong Hu, Xiaoju Li

The Funding statement for this article is incorrect. The correct Funding statement is as follows: This work was supported by the 2022 Undergraduate Quality and Teaching Reform Engineering Project of Guangdong Province (Advanced Manufacturing Experimental Teaching Demonstration Center) funded by the Department of Education of Guangdong Province (Grant Number: 2022zlgc006).
