# Supplementary material for: Research on fire scenario analysis and emergency response strategies for L-shaped buildings using FDS
Source: PLoS One. 2026 Apr 13;21(4):e0346927. doi: 10.1371/journal.pone.0346927 (PMC13075666; doi:10.1371/journal.pone.0346927)
Supplement: Attachment — Submitted filename: Response to reviewers.docx [file pone.0346927.s002.docx]

We extend our gratitude to Academic Editor Dr Gianluca Genovese for the review comments on the manuscript entitled ‘Analysis of Fire Scenarios and Emergency Response Strategies for L-shaped Buildings Using FDS’ (Manuscript ID: PONE-D-25-55298). The principal revisions and our responses to the Academic Editor’s comments are as follows:

1. There are multiple instances in the text where reference links appear as “Error! Reference source not found.” Please ensure that all citations and cross-references are correctly formatted and correspond to the entries in the reference list.

Response: We have revised the formatting to ensure correctness.

2. Many figures in the manuscript appear very small, and their details — such as text labels, legends, and scales — are difficult to read.

Response: We have revised the image dimensions and details and uploaded them separately.
