# Supplementary material for: Research on fire scenario analysis and emergency response strategies for L-shaped buildings using FDS
Source: PLoS One. 2026 Apr 13;21(4):e0346927. doi: 10.1371/journal.pone.0346927 (PMC13075666; doi:10.1371/journal.pone.0346927)
Supplement: Attachment — Submitted filename: Review for PONE-D-25-63824.docx [file pone.0346927.s003.docx]

Article title -
Research on Fire Scenario Analysis and Emergency Response Strategies for Lshaped Buildings Using FDS

Abstract :

The abstract is broad and conceptual. It focus more on the “informatization” and “human-machine collaboration” without clearly stated the problem or issues that created as base to this research.

- The author can clearly state the practical problem or gap in current fire rescue or some prediction methods.

Introduction :

1. The introduction establishes a strong contextual background, linking urbanization, high-rise development, and increasing fire risk. However, the research objective is not explicitly and concisely stated.

- author may add a clear sentence defining the research objective at the end of the problem statement.

2. The topic is highly significant, addressing real-world challenges in fire safety, evacuation planning, and intelligent rescue systems. The introduction does not clearly articulate why L-shaped buildings represent a critical unresolved problem compared to other geometries.

- Authors need introduce L-shaped buildings as a distinct fire risk category earlier in the introduction.

Establishing the Fire Model:

The model adopts performance-based fire safety criteria, aligning with international best practices. Multiple fire scenarios are systematically constructed, allowing comparative analysis across different unfavourable conditions.

- Author can clearly state that existing fire simulation studies often focus on regular building layouts and lack scenario-based frameworks tailored to complex geometries such as L-shaped buildings.

The Influence of Building Parameters on Fire Spread:

1. The section demonstrates that small changes in building-related parameters lead to fundamentally different evacuation outcomes, which is a strong and policy-relevant finding.

2. L-shaped geometry is shown to create asymmetric smoke propagation, reinforcing the inadequacy of generic fire safety assumptions.

3. The linkage between fire spread patterns and dynamic evacuation decision-making (stairwell choice vs. rescue capsule deployment) is a notable applied contribution.

4. Section 3 does not clearly state how these findings directly inform the emergency response strategies claimed in the title until late in the section.

- The author may ;
  1. Align the Introduction with Section 3 Objectives

2. Emphasize Practical and Regulatory Relevance

3. Clarify the Contribution to Emergency Response Strategy Development

Conclusion :

The conclusion is moderate and need more amendment.

- The author need to explicitly state the study’s core contribution and emphasize key findings from the data analysis done.
