# Supplementary material for: Research on fire scenario analysis and emergency response strategies for L-shaped buildings using FDS
Source: PLoS One. 2026 Apr 13;21(4):e0346927. doi: 10.1371/journal.pone.0346927 (PMC13075666; doi:10.1371/journal.pone.0346927)
Supplement: Attachment — Submitted filename: Response_to_Reviewers_auresp_1.docx [file pone.0346927.s004.docx]

Dear Editor and Reviewers,

We thank the reviewers for providing constructive feedback. We have fully revised our manuscript（PONE-D-25-63824）and have addressed all of the reviewers’ comments, as well as added new analyses to further strengthen our work. The major revisions and new analyses we have undertaken are summarized below and discussed in detail in the point-by-point responses.

(Note: Added and amended content in the revised draft has been highlighted in blue， while the primary revisions in the revised draft have been highlighted in yellow.)

**Response to Editorial Comments**

Article title -
Research on Fire Scenario Analysis and Emergency Response Strategies for Lshaped Buildings Using FDS

**Abstract** :
The abstract is broad and conceptual. It focus more on the “informatization” and “human-machine collaboration” without clearly stated the problem or issues that created as base to this research.

- The author can clearly state the practical problem or gap in current fire rescue or some prediction methods.

Response:

We are most grateful for the editor's valuable comments. In accordance with your suggestions, we have carefully revised the manuscript. Specifically, the first paragraph of the abstract of the revised draft has been amended as follows:

Existing fire simulation models primarily focus on the impact of individual building parameters, lacking a systematic analysis of the multi-factor coupling effects on fire spread. As a result, it is difficult to accurately predict the trend of fire spread and meet the demands of dynamic escape routes for three-dimensional rescue systems.

**Introduction :**

1. The introduction establishes a strong contextual background, linking urbanization, high-rise development, and increasing fire risk. However, the research objective is not explicitly and concisely stated.

- author may add a clear sentence defining the research objective at the end of the problem statement.

Response:

We sincerely appreciate the editor's valuable comments. In accordance with your suggestions, the research objectives of this paper have been clarified in the introduction. The content is as follows:

(1) Third paragraph of the introduction of the revised manuscript:

To address these issues, this study proposes a multi-scenario coupled fire evolution simulation framework, taking L-shaped buildings as an example.

(2) Fourth paragraph of the introduction of the revised manuscript:

This study aims to build a multi-factor coupled fire evolution prediction model to forecast fire trends, providing a theoretical basis for generating spatial and dynamic escape routes of trapped individuals in different locations within L-shaped buildings, thereby improving rescue efficiency for trapped individuals.

2. The topic is highly significant, addressing real-world challenges in fire safety, evacuation planning, and intelligent rescue systems. The introduction does not clearly articulate why L-shaped buildings represent a critical unresolved problem compared to other geometries.

- Authors need introduce L-shaped buildings as a distinct fire risk category earlier in the introduction.

Response:

We sincerely appreciate the editor's valuable comments. In accordance with your suggestions, the introduction now includes L-shaped buildings as a distinct fire risk category. The specific amendment is located in the fourth paragraph of the introduction of the revised draft, as follows:

L-shaped buildings are common representative asymmetric structures, with their corner structures easily creating special airflow patterns that affect the spread of smoke. This study aims to build a multi-factor coupled fire evolution prediction model to forecast fire trends, providing a theoretical basis for generating spatial and dynamic escape routes of trapped individuals in different locations within L-shaped buildings, thereby improving rescue efficiency for trapped individuals.

**Establishing the Fire Model:**

The model adopts performance-based fire safety criteria, aligning with international best practices. Multiple fire scenarios are systematically constructed, allowing comparative analysis across different unfavourable conditions.

- Author can clearly state that existing fire simulation studies often focus on regular building layouts and lack scenario-based frameworks tailored to complex geometries such as L-shaped buildings.

Response:

We sincerely appreciate the editor's valuable feedback. In accordance with your suggestions, contextual content has been incorporated. Specific revisions are detailed in the introduction and the Establishing the Fire Model section of the revised manuscript, as follows:

The introduction now includes: "L-shaped buildings are common representative asymmetric structures, with their corner structures easily creating special airflow patterns that affect the spread of smoke. This study aims to build a multi-factor coupled fire evolution prediction model to forecast fire trends, providing a theoretical basis for generating spatial and dynamic escape routes of trapped individuals in different locations within L-shaped buildings, thereby improving rescue efficiency for trapped individuals."

In the Establishing the Fire Model section, we have added: "Existing fire simulation studies primarily focus on regular building layouts, lacking scene-based frameworks for complex geometries such as L-shaped buildings. To reveal the fire evolution patterns in L-shaped buildings and quantify the coupling effects of three key factors— initial fire locations (stairwell or corridor corner), floor height of ignition (low/mid/high area), and wind direction—on fire spread inside the building and along exterior walls, this study establishes a fire model for a 15-story L-shaped building."

“Following the worst-case scenario principle, fire source locations were selected at representative positions anticipated to yield the most rapid fire spread and severe hazards. A fire originating on the ground floor, which features multiple exits and serves as a critical evacuation path, can propagate swiftly. A fire on a middle floor may hinder evacuation on both upper and lower levels, while a fire on the top floor is highly susceptible to the stack effect, complicating spatial rescue operations. Consequently, for the 15-story high-rise building under investigation, three representative floors were designated as ignition levels: the ground floor (1st floor), a middle floor (8th floor), and the top floor (15th floor).”

**The Influence of Building Parameters on Fire Spread:**
1. The section demonstrates that small changes in building-related parameters lead to fundamentally different evacuation outcomes, which is a strong and policy-relevant finding.

2. L-shaped geometry is shown to create asymmetric smoke propagation, reinforcing the inadequacy of generic fire safety assumptions.

3. The linkage between fire spread patterns and dynamic evacuation decision-making (stairwell choice vs. rescue capsule deployment) is a notable applied contribution.

4. Section 3 does not clearly state how these findings directly inform the emergency response strategies claimed in the title until late in the section.

- The author may ;

1. Align the Introduction with Section 3 Objectives

Response:

We sincerely appreciate the editor's valuable feedback. In accordance with your suggestions, we have carefully revised the manuscript to ensure consistency between the introduction and the section entitled 'The Influence of Building Parameters on Fire Spread'(Section 3)'s objectives. The specific modifications are as follows:

The introduction now emphasises the research objective: "This study aims to build a multi-factor coupled fire evolution prediction model to forecast fire trends, providing a theoretical basis for generating spatial and dynamic escape routes of trapped individuals in different locations within L-shaped buildings, thereby improving rescue efficiency for trapped individuals."

The section entitled 'The Influence of Building Parameters on Fire Spread'(Section 3) has been amended to state: "Based on the above analysis, it can be concluded that the L-shaped building geometry induces asymmetric smoke dispersion, and fire spread is the result of the coupled effects of multiple on-site factors. Safe evacuation decisions are directly correlated with the location of trapped occupants, the time of alarm activation, and the rescue method employed. Minor variations in any parameter can lead to fundamental differences in evacuation outcomes. Existing generic, static fire safety evacuation guidance demonstrates significant limitations. Consequently, generating real-time, dynamic escape paths based on actual fireground conditions is essential for improving rescue efficiency and success rates."

2. Emphasize Practical and Regulatory Relevance

3. Clarify the Contribution to Emergency Response Strategy Development

Response:

The specific amendments addressing the aforementioned two issues include revisions to the final paragraph of the section entitled 'The Influence of Building Parameters on Fire Spread' of the revised draft, as detailed below:

Based on the above analysis, it can be concluded that the L-shaped building geometry induces asymmetric smoke dispersion, and fire spread is the result of the coupled effects of multiple on-site factors. Safe evacuation decisions are directly correlated with the location of trapped occupants, the time of alarm activation, and the rescue method employed. Minor variations in any parameter can lead to fundamental differences in evacuation outcomes. Existing generic, static fire safety evacuation guidance demonstrates significant limitations. Consequently, generating real-time, dynamic escape paths based on actual fireground conditions is essential for improving rescue efficiency and success rates.

**Conclusion :**

The conclusion is moderate and need more amendment.

- The author need to explicitly state the study’s core contribution and emphasize key findings from the data analysis done.

Response:

We are most grateful for the editor's valuable comments. In accordance with your suggestions, we have carefully revised the manuscript to clearly articulate the core contributions of the research and highlight the key findings derived from the data analysis. Specific modifications include the first paragraph of the Conclusions section of the revised manuscript:

This study established a fire simulation model based on the Fire Dynamics Simulator (FDS) for a 15-story L-shaped building. Numerical simulations were conducted for representative scenarios under worst-case fire conditions, considering various combinations of initial fire location, floor height of ignition, and wind direction. The analysis systematically revealed the dynamic spread patterns and evolutionary characteristics of smoke within the fire floor, inside stairwells across different floors, and along the building's external facade. The innovation and core contributions of this research are primarily manifested in the following aspects. First, a multi-factor coupled fire evolution prediction model for L-shaped buildings was developed. This model elucidates the fire development patterns in such structures and clarifies the relationship between fire spread modes and dynamic evacuation decision-making. It not only provides a theoretical basis for generating dynamic escape paths for occupants trapped in different building locations but also lays a scientific foundation for the intelligent dispatch and deployment of emergency rescue systems. Second, "visibility" was explicitly identified as the core criterion for determining occupant safe evacuation in fire scenarios. This addresses limitations in parameter selection inherent in traditional risk assessments and offers a more reliable technical basis for the quantitative evaluation of fire risk and the refinement of emergency decision-making. Third, a generalized, multi-scenario coupled fire evolution simulation framework was proposed. This framework transcends the application constraints associated with a single building type, providing significant scientific support for the formulation, optimization, and engineering implementation of fire safety standards. The findings of this study can be directly applied to fire emergency response operations in actual buildings. A key direction for future research involves integrating the simulation data obtained from the worst-case fire scenarios in this study with machine learning techniques. This integration aims to construct a spatiotemporal database of building-wide evacuation patterns and to achieve the rapid generation of real-time, dynamic escape routes.

**Response to Reviewer 1's Comments**

The manuscript presents a comprehensive numerical investigation of fire scenario evolution and emergency response strategies in an L-shaped high-rise building using FDS. The study addresses a relevant and practical fire safety problem, particularly for complex building geometries that are often underrepresented in existing fire modeling literature. The systematic construction of multiple fire scenarios and the identification of visibility as a key safety-critical parameter are valuable contributions.

However, while the technical execution of the simulations is generally sound, the manuscript would benefit from clearer articulation of its research objectives, stronger justification of modeling assumptions, and a more explicit positioning of its novelty and contribution relative to existing studies. Strengthening these aspects would significantly improve the clarity, scientific rigor, and impact of the work.

Response:

We sincerely appreciate the editor's valuable comments. In accordance with your suggestions, we have carefully revised the manuscript, with all modifications highlighted in blue.

1. Regarding the comment "clearer articulation of its research objectives" and “ a more explicit positioning of its novelty and contribution relative to existing studies” the specific revisions are as follows:

(1) First paragraph of the abstract on of the revised manuscript:

Existing fire simulation models primarily focus on the impact of individual building parameters, lacking a systematic analysis of the multi-factor coupling effects on fire spread. As a result, it is difficult to accurately predict the trend of fire spread and meet the demands of dynamic escape routes for three-dimensional rescue systems.

(2) Fourth paragraph of the Introduction of the revised draft:

This study aims to build a multi-factor coupled fire evolution prediction model to forecast fire trends, providing a theoretical basis for generating spatial and dynamic escape routes of trapped individuals in different locations within L-shaped buildings, thereby improving rescue efficiency for trapped individuals.

(3) Final paragraph of the section entitled 'The Influence of Building Parameters on Fire Spread ' of the revised draft:

Based on the above analysis, it can be concluded that the L-shaped building geometry induces asymmetric smoke dispersion, and fire spread is the result of the coupled effects of multiple on-site factors. Safe evacuation decisions are directly correlated with the location of trapped occupants, the time of alarm activation, and the rescue method employed. Minor variations in any parameter can lead to fundamental differences in evacuation outcomes. Existing generic, static fire safety evacuation guidance demonstrates significant limitations. Consequently, generating real-time, dynamic escape paths based on actual fireground conditions is essential for improving rescue efficiency and success rates.

(4) First paragraph of the Conclusions section of the revised draft:

This study established a fire simulation model based on the Fire Dynamics Simulator (FDS) for a 15-story L-shaped building. Numerical simulations were conducted for representative scenarios under worst-case fire conditions, considering various combinations of initial fire location, floor height of ignition, and wind direction. The analysis systematically revealed the dynamic spread patterns and evolutionary characteristics of smoke within the fire floor, inside stairwells across different floors, and along the building's external facade. The innovation and core contributions of this research are primarily manifested in the following aspects. First, a multi-factor coupled fire evolution prediction model for L-shaped buildings was developed. This model elucidates the fire development patterns in such structures and clarifies the relationship between fire spread modes and dynamic evacuation decision-making. It not only provides a theoretical basis for generating dynamic escape paths for occupants trapped in different building locations but also lays a scientific foundation for the intelligent dispatch and deployment of emergency rescue systems. Second, "visibility" was explicitly identified as the core criterion for determining occupant safe evacuation in fire scenarios. This addresses limitations in parameter selection inherent in traditional risk assessments and offers a more reliable technical basis for the quantitative evaluation of fire risk and the refinement of emergency decision-making. Third, a generalized, multi-scenario coupled fire evolution simulation framework was proposed. This framework transcends the application constraints associated with a single building type, providing significant scientific support for the formulation, optimization, and engineering implementation of fire safety standards. The findings of this study can be directly applied to fire emergency response operations in actual buildings. A key direction for future research involves integrating the simulation data obtained from the worst-case fire scenarios in this study with machine learning techniques. This integration aims to construct a spatiotemporal database of building-wide evacuation patterns and to achieve the rapid generation of real-time, dynamic escape routes.

2.In response to the suggestion to “ stronger justification of modeling assumptions” the specific modifications are as follows:

(1) First and second paragraphs of the Fire Scenario Parameter Settings section of the revised draft:

Following the worst-case scenario principle, fire source locations were selected at representative positions anticipated to yield the most rapid fire spread and severe hazards. A fire originating on the ground floor, which features multiple exits and serves as a critical evacuation path, can propagate swiftly. A fire on a middle floor may hinder evacuation on both upper and lower levels, while a fire on the top floor is highly susceptible to the stack effect, complicating spatial rescue operations. Consequently, for the 15-story high-rise building under investigation, three representative floors were designated as ignition levels: the ground floor (1st floor), a middle floor (8th floor), and the top floor (15th floor).

The corridor outside the room nearest to staircases or the building corner was identified as a critical segment of the evacuation path. An ignition point near a staircase could compromise its functionality for egress, whereas a point near a corner could be significantly influenced by local ventilation conditions. Accordingly, three representative ignition points were defined: Initial fire location ① in Room 13, closest to Staircase 1; Initial fire location ② in Room 17, nearest to the L-shaped building corner; and Initial fire location ③ in Room 22, closest to Staircase 2. These three points, marked by red squares "■" in Fig 1(a), were utilized to investigate the impact of fire source location on fire spread dynamics.

(2) Fourth and fifth paragraphs of the Fire Scenario Parameter Settings section of the revised draft:

Based on the typical heat release rate for office fires (GB 51251-2017), the fire source was set to a constant heat release rate of 1.5 MW. To simulate the fire resistance characteristics of reinforced concrete structures meeting the Chinese Grade II fire resistance rating requirements (GB 50016-2014), building surface materials were specified as inert, with a 0.1 m thick gypsum layer. The combustion reaction was modeled on polyurethane, a representative flammable material found in offices.

Given that the multi-year average wind speed at a 10 m height in Guangzhou, China is 1.9 m/s, which reflects the regional wind environment, a velocity (V0) of 1.9 m/s at a reference height (H0) of 10 m was adopted for the simulations.

**Response to Reviewer 2's Comments**

This paper presents a research on the dynamic evolution of smoke dispersion under varying conditions—including initial fire locations (stairwells or corridor bends), floor height of ignition (low/medium/high zones), and wind direction. However, the description of the abstract needs to be redesigned.

1. There has been extensive research on building fires. What is the most important innovation point in this paper?

Response:

The most important innovation point in this pape is summarised in the first paragraph of the Conclusions section of the revised manuscript. The specific revisions are as follows:

The innovation and core contributions of this research are primarily manifested in the following aspects. First, a multi-factor coupled fire evolution prediction model for L-shaped buildings was developed. This model elucidates the fire development patterns in such structures and clarifies the relationship between fire spread modes and dynamic evacuation decision-making. It not only provides a theoretical basis for generating dynamic escape paths for occupants trapped in different building locations but also lays a scientific foundation for the intelligent dispatch and deployment of emergency rescue systems. Second, "visibility" was explicitly identified as the core criterion for determining occupant safe evacuation in fire scenarios. This addresses limitations in parameter selection inherent in traditional risk assessments and offers a more reliable technical basis for the quantitative evaluation of fire risk and the refinement of emergency decision-making. Third, a generalized, multi-scenario coupled fire evolution simulation framework was proposed. This framework transcends the application constraints associated with a single building type, providing significant scientific support for the formulation, optimization, and engineering implementation of fire safety standards.

2. What is the basis for setting fire scene parameters?

Response:

The basis for setting fire scene parameters has been added, with specific modifications as follows:

(1) First and second paragraphs of the Fire Scenario Parameter Settings section of the revised draft:

Following the worst-case scenario principle, fire source locations were selected at representative positions anticipated to yield the most rapid fire spread and severe hazards. A fire originating on the ground floor, which features multiple exits and serves as a critical evacuation path, can propagate swiftly. A fire on a middle floor may hinder evacuation on both upper and lower levels, while a fire on the top floor is highly susceptible to the stack effect, complicating spatial rescue operations. Consequently, for the 15-story high-rise building under investigation, three representative floors were designated as ignition levels: the ground floor (1st floor), a middle floor (8th floor), and the top floor (15th floor).

The corridor outside the room nearest to staircases or the building corner was identified as a critical segment of the evacuation path. An ignition point near a staircase could compromise its functionality for egress, whereas a point near a corner could be significantly influenced by local ventilation conditions. Accordingly, three representative ignition points were defined: Initial fire location ① in Room 13, closest to Staircase 1; Initial fire location ② in Room 17, nearest to the L-shaped building corner; and Initial fire location ③ in Room 22, closest to Staircase 2. These three points, marked by red squares "■" in Fig 1(a), were utilized to investigate the impact of fire source location on fire spread dynamics.

(2) Fourth and fifth paragraphs of the Fire Scenario Parameter Settings section of the revised draft:

Based on the typical heat release rate for office fires (GB 51251-2017), the fire source was set to a constant heat release rate of 1.5 MW. To simulate the fire resistance characteristics of reinforced concrete structures meeting the Chinese Grade II fire resistance rating requirements (GB 50016-2014), building surface materials were specified as inert, with a 0.1 m thick gypsum layer. The combustion reaction was modeled on polyurethane, a representative flammable material found in offices.

Given that the multi-year average wind speed at a 10 m height in Guangzhou, China is 1.9 m/s, which reflects the regional wind environment, a velocity (V0) of 1.9 m/s at a reference height (H0) of 10 m was adopted for the simulations.

3. Numerical simulations lack verification of grid independence.

Response:

Thank you for your valuable feedback. In order to select the appropriate mesh, we have conducted simulation comparisons. Case 1—defined by ignition in Room 13 adjacent to Stairwell 1 on the 8th floor (mid-level)—was selected to simulate temperature changes in Corridor Area A  (whose measurement point is was closest to the initial fire location) under different grid configurations.As shown in Fig 1, a grid size of 0.5m × 0.5m × 0.5m achieved the maximum grid scale while ensuring the computational results remain grid-independent. Taking into account the dual considerations of simulation accuracy and computational efficiency, a uniform grid size of 0.5 m × 0.5 m × 0.5 m was selected as the design value for the computational model and was adopted for all subsequent numerical simulations and experimental investigations. Owing to the limited scope of this paper and the fact that network irrelevance is not the primary focus, the following content has been added to the Fire Scenario Parameter Design section:

Taking into account the dual considerations of simulation accuracy and computational efficiency, a uniform grid size of 0.5 m × 0.5 m × 0.5 m was selected as the design value for the computational model and was adopted for all subsequent numerical simulations and experimental investigations.

Fig 1 Temperature-over-time curve for Corridor Area A under different grid configurations

4. The article's expression is not standardized and there are some incorrect expressions.

The language description needs improvement.

Response:

Based on the reviewers' comments, we have carefully refined the manuscript and corrected the grammatical, stylistic, and spelling errors identified in the manuscript. The primary revisions in the revised draft have been highlighted in yellow.

We would like to express our sincere gratitude to the editors and reviewers for their valuable feedback on our paper. Thank you!

Sincerely,

Qin Zhang

Yuhong Hu

Xiaoju Li

Corresponding author: Qin Zhang (E-mail: zhangqin@dgcu.edu.cn)

February 14, 2026
